# Can Gut Microbiota Composition Predict Response to Dietary Treatments?

**DOI:** 10.3390/nu11051134

**Published:** 2019-05-22

**Authors:** Jessica R Biesiekierski, Jonna Jalanka, Heidi M Staudacher

**Affiliations:** 1 Department of Dietetics, Nutrition & Sport, School of Allied Health Human Services & Sport, La Trobe University, 3086 Melbourne, Australia; 2 Human Microbiome Research Program, Faculty of Medicine, University of Helsinki, 00014 HY Helsinki, Finland; jonna.jalanka@helsinki.fi; 3 Nottingham Digestive Diseases Centre and NIHR Nottingham Biomedical Research Centre at Nottingham University Hospitals NHS Trust, the University of Nottingham, Nottingham NG7 2RD, UK; 4 Food and Mood Centre, IMPACT SRC, Deakin University, Geelong, VIC 3216, Australia; heidi.staudacher@deakin.edu.au

**Keywords:** personalised nutrition, microbiota, dietary intervention, obesity, irritable bowel syndrome, gastrointestinal symptoms

## Abstract

Dietary intervention is a challenge in clinical practice because of inter-individual variability in clinical response. Gut microbiota is mechanistically relevant for a number of disease states and consequently has been incorporated as a key variable in personalised nutrition models within the research context. This paper aims to review the evidence related to the predictive capacity of baseline microbiota for clinical response to dietary intervention in two specific health conditions, namely, obesity and irritable bowel syndrome (IBS). Clinical trials and larger predictive modelling studies were identified and critically evaluated. The findings reveal inconsistent evidence to support baseline microbiota as an accurate predictor of weight loss or glycaemic response in obesity, or as a predictor of symptom improvement in irritable bowel syndrome, in dietary intervention trials. Despite advancement in quantification methodologies, research in this area remains challenging and larger scale studies are needed until personalised nutrition is realistically achievable and can be translated to clinical practice.

## 1. Introduction

Diet is a modifiable risk factor for many non-communicable diseases and there is a high level of evidence supporting the efficacy of dietary interventions for both influencing disease risk and improving disease outcomes. For example, dietary intervention can reduce cardiovascular disease risk by 60% [1] and can successfully reduce gastrointestinal (GI) symptoms in at least 50% of patients with irritable bowel syndrome (IBS) [2]. However, individual variability in response to treatment is increasingly recognised, and this is reflected in the highly variable response rates in clinical trials of dietary interventions, particularly in obesity [3], cardiovascular disease [4] and IBS [2].

Personalised nutrition essentially enables the tailoring of dietary advice to the individual level through the incorporation of data related to specific biological pathways driving that individual’s health or disease status, ultimately optimising the effectiveness of the advice. A comprehensive understanding of clinical conditions and underlying disease mechanisms is often required, including the genetic variants of the patient and the extent to which these variants interact with diet to affect disease risk and treatment in diverse populations.

Personalised nutrition models integrate a variety of host-specific variables including current diet, biological or phenotypical characteristics of the individual (age, stage of life, gender, body mass index (BMI), disease or health status) and genotypic characteristics. An understanding of epigenetics (regulation of gene expression) is also often included. Models will vary with the clinical condition and its underlying mechanisms, and with the research hypothesis, where different combinations of characteristics are possible. Most evidence supporting personalised nutrition has come from observational studies with disease risk factors as outcomes (e.g., postprandial glucose response). However, there are some trials using clinical endpoints that have incorporated participant information to test prediction of responses [5] and large-scale studies collecting multi-dimensional data to predict response to acute diet challenges [6].

Gut microbiota is one variable shown to be mechanistically relevant for a number of disease states and therefore has a potential role in the development of personalised dietary advice. Microbiota has a profound impact on our health, and alterations to its composition and its dysfunction have been associated with several chronic diseases [7]. Despite the lack of a clear definition of a “healthy microbiota”, its general hallmarks include its resistance to compositional change and its responsiveness to environmental challenges [8]. This allows continuous operation of essential metabolic and immune functions including host nutrient metabolism, maintenance of structural integrity of the gut mucosal barrier, immunomodulation and protection against pathogens.

Diet is well known as one of the major drivers of microbiota composition [9] and conversely, the microbiota response to dietary intervention varies between individuals [10]. In the last decade, efforts have been directed to beginning to understand how biological response may be influenced by the baseline microbiota [11]. Much of the research investigating the predictive capacity of baseline microbiota for clinical response to dietary intervention has been reported in two specific health conditions, namely, obesity and IBS.

### 1.1. Clinical Condition 1: Obesity

Overweight and obesity rates continue to rise worldwide. In 2013, among adults (age ≥20 years), 37% of men and 30% of women were considered overweight (BMI 25–29.9) or obese (BMI ≥30) [12]. Obesity is associated with numerous chronic diseases and increases the risk for type 2 diabetes, metabolic disorder and cancer [13,14]. The pathogenesis of obesity is complex, with environmental, sociocultural, behavioural, physiological, genetic and epigenetic factors known to be contributors. Treatment often requires significant behaviour modification including dietary change and physical activity. Common dietary approaches include a low-fat diet, a high-protein diet or the DASH (Dietary Approaches to Stop Hypertension) diet [15,16]. Pharmacotherapy, medical devices and bariatric surgery are other treatment options for patients requiring additional intervention. Given the multifaceted nature of obesity, there is no single nor simple treatment solution, and therefore novel, and most likely personalised, interventions may be necessary for effective treatment.

### 1.2. Clinical Condition 2: Irritable Bowel Syndrome

Irritable bowel syndrome (IBS), a chronic functional bowel disorder characterised by abdominal pain and altered bowel habit [17], affects 11% of individuals globally [18]. Positive diagnosis is based on the symptom profile meeting Rome IV criteria, and patients are classified into one of four IBS subtypes (diarrhoea-predominant, constipation-predominant, mixed and unsubtyped) [17]. The pathophysiology of IBS is not completely elucidated. Most factors proposed are embodied with the concept of a disturbed bidirectional brain–gut axis, including alterations in the central nervous system (e.g., high prevalence of anxiety and depression), visceral hypersensitivity, increased gut epithelial permeability, low grade inflammation and an altered microbiome.

Lifestyle advice, including healthy diet and exercise, is usually considered as first line therapy, followed by symptom-directed pharmacotherapies (anti-spasmodics, laxatives, pro-secretory agents) which have varying efficacy and safety profile [19]. The low FODMAP diet, an approach restricting the intake of specific fermentable carbohydrates (i.e., oligo-, di-, mono-saccharides and polyols) is a second-line dietary intervention [20] and, although successful in inducing global symptom response in many, is often not effective for up to 50% of patients [2].

### 1.3. Purpose of Review

Recent research suggests that dietary advice could be revolutionised towards a more personalised approach for a spectrum of disease states. Accurate prediction of clinical response, such as weight loss in obesity or symptom improvement in IBS, may not only improve short-term clinical response but also long-term treatment efficacy and overall health outcomes in response to dietary intervention. This paper aims to review the current state of evidence related to how knowledge of gut microbiota may facilitate personalised dietary treatments in obesity and in IBS, with a focus on human dietary intervention trials. In addition, the article aims to identify the knowledge gaps and address the implications of research to date. Studies were selected for inclusion if they assessed baseline microbiota composition as a prediction tool for the clinical response after dietary intervention in human cohorts with IBS and obesity. Can gut microbiota composition predict response to dietary treatments?

### 1.4. Role of Intestinal Microbiota in Obesity

Several lines of evidence support a role for microbiota in the pathophysiology of obesity. The obese mouse model is characterised by a 50% decrease in *Bacteroidetes* abundance, an increase in *Firmicutes* [21] and a lower abundance of *Akkermansia muciniphila* [22] compared with lean mice. Clinical research in humans supports this, with evidence of fewer *Bacteroides* and more *Firmicutes* [21], as well as a lower abundance of *Bifidobacteria* [23] compared with lean controls. Obesity is also associated with a lower bacterial richness (where richness is defined as the number of different species in an ecosystem) and those with a lower bacterial richness gain more weight over time [24]. Efficacy of probiotics [25] and faecal microbiota transplantation (FMT) [26] (via colonisation with “lean microbiota”) to induce weight loss in obese individuals implies that attempts at “correcting” the microbial equilibrium can influence body weight and adiposity in obesity.

Although the underlying mechanisms by which gut microbiota contributes to obesity are not fully understood, evidence suggests contributing pathways include activity of the fermentation by-product short-chain fatty acids (SCFAs) in regulating gut hormones and influencing energy harvest [27]. Gut microbiota may also suppress the production of fasting-induced adipose factors [28] and be linked to inflammatory responses [29], regulation of lipogenesis pathways of triglyceride synthesis [30] and impaired innate immune interactions [31].

### 1.5. Impact of Dietary Treatment on the Microbiome in Obesity

Energy restriction is the staple dietary intervention in obesity. When obese humans are assigned to either a fat-restricted or carbohydrate-restricted diet, the resulting increase in abundance of *Bacteroidetes* correlates with percentage loss of body weight [21]. Others demonstrate that three months of a formula-based very low-calorie diet (800 kcal/day) in 18 obese adults led to 21 kg average weight loss with concomitant changes in microbiota and bacterial metabolism [32]. The indicative taxa for the microbial diversity change involved the increase in *Acinetobacter.* Furthermore, a six-week energy-restricted high-protein diet in 38 obese adults improved low gene richness (i.e., number of detected bacterial genes) and increased the abundance of most gene clusters [33]. Another study assessed the impact of a Mediterranean diet compared with a low-fat diet in 20 obese men. There were no significant differences in the metabolic variables measured (weight change was not reported) between the diets after one year of dietary intervention. However, the low-fat diet group demonstrated an increased relative abundance of *Prevotella* and decreased *Roseburia* genera from baseline, whereas the Mediterranean diet led to the reverse, a decreased abundance of *Prevotella* and increased abundances of the *Roseburia* and *Oscillospira* genera from baseline [34]. These diets led to differential alterations in gut microbiota due to changes in food groups.

In addition to energy restriction, dietary interventions designed to target gut microbiota have used a range of potential modulators and have assessed various obesity risk factors. For example, one prebiotic supplementation study showed that a 16-week intervention of oligofructose-enriched inulin in overweight or obese children (7–12 years) led to a greater abundance of *Bifidobacteria* compared with controls who received maltodextrin [35]. Many other within-group changes were observed for microbiota, but importantly, changes in gut microbial abundance coincided with beneficial changes in body composition and biological parameters of interleukin-6 and serum triglycerides compared with controls. Others have studied the effect of prebiotics through food or whole diet interventions. One uncontrolled trial delivered a diet rich in non-digestible carbohydrates (based on whole grains, traditional Chinese medicinal foods and prebiotics) via hospitalised intervention in 21 morbidly obese children (3–16 years) for 30 days [36]. Microbiota composition, which had been enriched with potentially pathogenic bacteria at baseline, was much higher post-intervention in beneficial groups of bacteria, such as *Bifidobacterium* spp. Structural changes at the individual bacterial genome level were also significantly associated with improvements in host metabolic health (e.g., serum antigen load, alleviation of inflammation) alongside the 9.5% loss of initial bodyweight [36].

### 1.6. Microbiome as a Predictor for Dietary Treatment Response in Obesity

In the last five years, there have been four relatively small dietary trials that assessed the association of baseline microbiota with either weight response in obese cohorts or the response of postprandial hyperglycaemia in healthy individuals, an independent risk factor for obesity. Furthermore, there have been two studies that used microbiota as a prediction tool to model weight response in obesity cohorts and two to predict glycaemic response in healthy individuals (Table 1).

First, a summary of the studies assessing weight response in obesity is presented. Two unrandomised trials have demonstrated the utility of baseline microbiota in predicting bodyweight response to dietary intervention. One showed that a higher gene richness at baseline was associated with a greater reduction in adipose tissue and systemic inflammation after a six-week energy-restricted high-protein diet (*n* = 38) [33]. The other reported that higher baseline abundance of *Akkermansia muciniphila* was associated with greater improvement in insulin sensitivity and body fat distribution after a six-week energy-restricted diet (*n* = 49) [37]. The first of two modelling studies implemented a six-week energy-restricted, high-protein diet followed by a six-week period of weight maintenance in obese or overweight individuals (*n* = 50) [38]. A combination of biological, gut microbiota and environmental factors were used to predict individual weight loss trajectory using a graphical Bayesian network framework. Those who lost the least weight and regained the most were characterised by higher abundances of *Lactobacillus*, *Leuconostoc and Pediococcus* genera. The overall microbiota composition at baseline was not identified by the framework as a predictor for weight loss. In another modelling study, the likelihood of weight loss in 78 obese adults undertaking high-fibre dietary interventions was related to the abundance of *Firmicutes* at baseline [39].

Key studies in the personalised nutrition field have investigated postprandial glycaemic responses (PPGR) to dietary intervention. One crossover randomised clinical trial (RCT) randomised 39 healthy participants to a three-day intervention of barley kernel-based bread or white wheat flour bread (100 g starch/day). The 10 participants demonstrating the most pronounced improvement in glucose and insulin response after a standardised breakfast following the barley kernel-based bread intervention were classified as responders. Responders were characterised by a higher *Prevotella/Bacteroides* ratio, higher relative abundance of *Dorea* and greater microbial potential to ferment complex oligosaccharides at baseline compared with non-responders [40]. Also implementing a bread intervention, a second RCT provided 20 healthy participants with three portions of 145 g sourdough-leavened whole-grain bread or 110 g white bread per day for one week. The interpersonal variability in glycaemic response to the different bread types could be reliably predicted with baseline microbiome data (accuracy ROC curve of 0.83) [41].

The most impressive study that has investigated predictors of biological responses to diet to date is a large predictive modelling study. Participants’ (*n* = 800) blood glucose was continuously monitored for one week whilst they recorded daily activities and dietary intake [6]. The PPGR to the first meal of every day, which was one of four different standardised meals (equivalent to 50 g carbohydrate), was a key component of a large dataset of clinical, anthropometric, dietary and biological information. PPGR variability was associated with a variety of clinical factors (HbA1c%, BMI, systolic blood pressure and alanine aminotransferase (ALT) activity). However, intriguingly, Proteobacteria and Enterobacteriaceae were positively associated with some of the PPGR to the standardised meal; associations with glycaemic response were also evident for certain microbial pathways at the functional level. From these data, a prediction model using thousands of decision trees based on 137 features representing meal content, daily activity, blood parameters and microbiome features was then validated in a separate 100-person cohort [6]. Similarly, others have used six-day PPGR data in 327 healthy participants to demonstrate that baseline microbiome combined with other physiological characteristics is highly predictive of postprandial responses. This was more predictive than standard clinical approaches that incorporate calorie or carbohydrate content alone [42].

Together, the findings from the obesity cohorts and glycaemic response studies highlight that microbiota composition and abundance of specific taxa present an exciting opportunity to enable predicting responsiveness to diet. However, challenges in interpreting the evidence exist, including the heterogeneity of studies, such as the length of dietary challenge (three to six weeks in obesity, three days to one week in PPGR), the type and intensity of intervention (level of caloric restriction, types of carbohydrate) and the number of taxa analysed. Most studies defined their subject cohort, including presence of comorbidity and medication use; however, many did not report on other external factors that could influence microbiota composition (e.g., probiotics), which may have contributed to the heterogeneity of findings.

### 1.7. Role of Intestinal Microbiota in IBS

Evidence for microbiota in the development and/or as a driving force of symptom severity in IBS has been accumulating for some 35 years. The line of evidence that is most well supported in the literature is the observation that the faecal microbiota composition of patients differs from that of healthy controls, although there is little consistency in findings across studies [43,44]. Reported differences include a higher *Firmicutes/Bacteroidetes* ratio [45,46,47,48,49], a lower *Bifidobacteria* abundance [48,50,51,52,53,54,55] and instability in response to dietary change [56] in IBS compared with healthy controls. Differences have not been limited to the luminal compartment; colonic mucosal microbial composition also deviates from healthy controls [46,51,57]. Distinct microbial profiles associated with severity of gut symptoms [47,48,49,57,58] or presence of psychological morbidity [47,57,58,59] support a potential role for microbiota in perpetuating symptoms. Although one of the most recent investigations of microbiota in IBS identified a distinct faecal and mucosal signature associated with categories of symptom severity in IBS [49], a unique and consistent microbial signature differentiating IBS from non-IBS has not been identified.

A number of other lines of evidence supporting the important role of microbiota in the pathophysiology of IBS come from animal models. The presence of transplanted microbiota from individuals with IBS in germ-free mice leads to the transfer of the disease state, including altered microbiota composition, visceral hypersensitivity, altered transit, immune activation and behavioural manifestations of the condition [60,61]. Other animal data suggest microbial metabolites, such as SCFAs, induce visceral hypersensitivity, a key feature of IBS [62]. In humans, additional support for the involvement of microbiota in IBS aetiology is the presence of systemic and mucosal immune activation, with the altered gut microbiota a potential key driver of this dysregulation [63]. Finally, although it is still unclear whether a divergent microbiota is a primary phenomenon, the efficacy of therapies such as probiotics and FMT implies that attempts at “correcting” the abnormality lead to at least partial restoration of microbial and GI equilibrium [64,65].

### 1.8. Impact of Dietary Treatment on the Microbiome in IBS

Over the past 10 years, clinical trials of the low FODMAP diet have vastly outnumbered studies of other ”whole diet” interventions in IBS. Response rates of 50–80% are reported in RCTs in which the advice is dietitian-led [2]. The impact of short-term FODMAP restriction on the faecal microbiota has been reported in a number of trials of dietary advice in free-living individuals with IBS [65,66,67,68,69] and in a highly controlled feeding trial [70]. A variety of taxonomic changes have been reported, of which the most consistent finding is a lower relative or absolute abundance of *Bifidobacteria* compared with controls and/or pre-intervention [65,66,67,69,70,71]. Interestingly, those individuals with greater *Bifidobacteria* abundance at baseline exhibit the greatest depletion in response to FODMAP restriction [65]. Altered metabolomic profile in response to FODMAP restriction has also been reported in IBS, suggesting there is a change in metabolic activity of microbes in response to reduced availability of fermentable carbohydrates or increased availability of alternative dietary substrates [67,71]. Whether these microbial changes are key to inducing symptomatic response to a low FODMAP diet is not known from the current evidence. The anti-bifidogenic effect of the low FODMAP diet is inconsistent with a “more is better” hypothesis that could be postulated from the inverse correlation between *Bifidobacteria* and symptom severity [48,57] and the trend toward efficacy of *Bifidobacteria*-containing probiotic supplements in IBS [72].

### 1.9. Microbiome as a Predictor for Dietary Treatment Response in IBS

The ability to predict symptomatic response to the low FODMAP diet has been of recent interest, particularly considering the diet is intensive to implement and requires dietetic supervision [73], and that up to half of individuals may not benefit. There is limited but consistent evidence that baseline demographic or clinical characteristics do not differentiate responders from non-responders to the low FODMAP diet [68,74,75,76].

Five studies have investigated whether baseline microbiota could predict symptomatic response to the low FODMAP diet (Table 2). One four-week RCT (19/33 responders, 61%) [5] and a four-week uncontrolled trial (32/61 responders, 52%) [77] of low FODMAP dietary advice propose baseline microbial profile to be predictive of response, using a microbial mapping technique based on selected DNA probes [78]. Of the 45 bacterial markers at baseline in the latter trial, 10 differentiated responders from non-responders with a positive predictive value of 76.0 (95% CI 61–87) using scores based on an arbitrary microbial “response index”. A third study, a small uncontrolled one-week trial in children (4/8 responders, 50%), reported that a lower abundance of saccharolytic *Bacteroides* and Bacteroidales was predictive of dietary response [68]. This was followed by a crossover RCT (8/33 responders, 24%) that reported baseline enrichment of a range of saccharolytic taxa including *Bacteroides* and *Faecalibacterium prausnitzii* in responders compared with non-responders [79]. However, not all studies report positive findings; one trial found no predictive value of baseline microbiota in determining clinical response to a low FODMAP diet, although the highly controlled four-week feeding RCT in adults (11/27 responders, 41%) based findings on abundances of a select few taxa using qPCR analysis. It must also be noted that both trials of crossover design may have influenced microbial composition of those receiving low FODMAP diet as the second intervention [68,70].

There are obvious challenges in interpreting the evidence from these trials. Pre-intervention environmental factors (e.g., medication, probiotic intake) that could impact on baseline microbiota composition are not always reported or controlled. Heterogeneity in baseline stool consistency and psychological comorbidity, both associated with altered microbiota composition [47,80,81], also complicate the identification of a distinct “responder” microbiota. Second, the stringency of low FODMAP dietary advice varies between studies. Insufficient FODMAP restriction could lead to an underestimation of true responders. Furthermore, clinical response criteria vary across studies, ranging from validated criteria [5,77] through to arbitrary cut-offs [70], leading to a sizable range of symptom severities in “responders” after low FODMAP treatment. Finally, no two studies so far have utilised the same statistical modelling techniques to explore the potential of microbiota as a predictor; ideally, the choice of statistical approach should be made by an independent blinded researcher to enable objective and statistically rigorous findings.

Based on the literature thus far, there is inconsistent evidence to support the use of one baseline microbiota signature to accurately predict response to a low FODMAP diet. Evidence that a baseline bacterial volatile organic compound profile may very accurately select responders [82] suggests the possibility that the metabolic function of bacteria may be important in determining response. Validation studies in well-controlled studies of specific IBS subtypes are warranted.

## 2. General Discussion and Limitations

Few human studies (summarised in Table 1 and Table 2) have been conducted investigating whether specific microbial signatures predict response to dietary interventions. The conditions of obesity and IBS represent the best examples of preliminary work conducted in this area. Based on this review, there is inconsistent evidence to support the existence of specific microbiota signatures to accurately predict clinical response to dietary intervention in obesity and IBS. A number of limitations still impede progress in this sphere of research.

First, microbial sampling (i.e., faecal or biopsy) and quantification methodologies applied across studies thus far have been inconsistent. The increasing power and sensitivity of modern sequencing techniques has led to the rapid development of high-throughput methods for assessing genome-wide genetic variations. However, the approaches used to characterise the human microbiota still vary widely. Furthermore, technical accuracy is crucial throughout processing and analysis. For example, the suboptimal mechanical lysis during extraction of the microbiota DNA from faecal or biopsy samples, a key step in the analysis pipeline [83], will distort the downstream analysis more than any other analysis step.

Second, there are several shortcomings in the predictive modelling analysis methods utilised. Therefore, it is important that consistent analysis pipelines be adopted worldwide enabling comparison of data between studies. Studies may be limited to exploratory statistical analysis until clinical studies can be adequately designed and powered for primary analysis.

Third, there are many problematic confounding factors that can impact on baseline microbiota composition. These factors include, but are not limited to, the host genetic makeup, long-term dietary habits, ethnicity, sanitation, geographical location, exercise and lifestyle habits, and antibiotic use. This further highlights the conclusion that any personalised predictive model incorporating gut microbial composition must consider multiple additional relevant individual datapoints, which may vary with disease state. It is also acknowledged that some chronic diseases, although benefiting from dietary intervention, may never be amenable to a microbiota-based personalised nutrition approach due to inherent heterogeneity in microbiota composition across individuals.

Finally, for ultimate translation into clinical practice, there is a need to understand if the results gained from short-term studies predicting host response can be translated into durable responses over time, leading to long-term positive health outcomes. Longer duration of studies and intervention periods are also needed.

## 3. Conclusions

Diet is one of the most important determinants of the gut microbiota composition. However, the relationship between diet and microbiota is complex and not completely understood. Consequently, personalised nutrition models that predict clinical response to dietary treatment based on the microbial composition are still extremely challenging to test in the research context. Some evidence of associations between gut microbiota and response to dietary treatments for both obesity and IBS suggests that links exist between microbiota composition and inter-individuality in host response to diet. However, personalised nutrition research is in its infancy and specific microbiota signatures that predict individualised responses to dietary treatment are still elusive; advancements in analysis technologies and consistent bioinformatic approaches will be important for progress.

## Figures and Tables

**Table 1:** Summary of recent trials reporting on the association between baseline gut microbiota composition and association with clinical response in obesity.

Study	Study Design	No. of Subjects	Clinical Condition	Dietary Intervention	Control	Key Findings	Ref.
**Controlled trials *–* weight response**							
Cotillard et al., 2013	Non-randomised clinical trial	49 (f = 41, m = 8)	Obesity (*n* = 38, and *n* = 11 overweight)	6-week energy-restricted high-protein diet followed by a 6-week weight-maintenance diet	-	Responders: Higher gene richness (where responders were those with marked improvement of adipose tissue and systemic inflammation).	[33]
Dao et al., 2016	Non-randomised clinical trial	49 (f = 41, m = 8)	Obesity and overweight	3-week calorie restriction	-	Responders: Higher gene richness and *Akkermansia muciniphila* abundance was associated with most improved body fat distribution, fasting plasma glucose, plasma triglycerides, improvement in insulin sensitivity.	[37]
**Modelling studies *–* weight response**							
Kong et al., 2013	Network modelling	50 (f = 42, m = 8)	Obesity and overweight	6-week energy-restricted, high-protein diet followed by maintenance phase	-	Responders: Baseline microbiota not identified as a predictor. Non-responders: High *Lactobacillus/Leuconostoc/Pediococcus*.	[38]
Korpela et al., 2014	Predictive modelling	78 (f = 40, m = 38)	3 cohorts with obesity	3 different types of dietary interventions varying in carbohydrate quality and quantity	-	Responders: High abundance of *Firmicutes*, where the microbiota composition was associated with change in serum cholesterol levels.	[39]
**Controlled trials *–* glycaemic response**							
Kovatcheva-Datchary et al., 2015	RCT, crossover	39 (f = 33, m = 6)	Healthy	3-day barley kernel-based bread	3-day white wheat flour bread	Responders: Higher *Prevotella/Bacteroides* ratio and increased *Dorea* that could predict PPGR to barley kernel-based bread.	[40]
Korem et al., 2017	RCT, crossover	20 (f = 11, m = 9)	Healthy	1 week of 3× 145 g whole-grain sourdough/day	1 week of 3× 110 g refined white bread/day	Responders: Specific microbial signature (especially abundances of *Coprobacter fastidiosus* and *Lachnospiraceae bacterium*) could predict PPGR to either bread.	[41]
**Modelling studies *–* glycaemic response**							
Zeevi et al., 2015	Machine learning algorithm	800 (f = 480, m = 320)	Healthy (assessing glycaemic response)	1-week usual diet with one standardised meal with 50 g available carbohydrate/day	-	Responders: Proteobacteria, Enterobacteriaceae and Actinobacteria were associated with elevated PPGRs. Non-responders: Clostridia and Prevotellaceae associated with lower PPGRs.	[6]
Mendes-Soares et al., 2019	Same modelling framework as Zeevi et al., 2015 [6]	327 (f = 255, m = 72)	Healthy (assessing glycaemic response)	6-day usual diet including four standardised meals with 50 g available carbohydrate	-	Baseline microbiota combined with other physiological characteristics was more predictive of PPGR than using only calorie or carbohydrate content of foods.	[42]

PPGR, postprandial glycaemic response; RCT, randomised clinical trial.

**Table 2:** Summary of recent trials reporting on the association between baseline gut microbiota composition and association with clinical response in IBS.

Study	Study Design	No. of Subjects	IBS Sub-Type	Dietary Intervention	Control	Key Findings	Ref.
Chumpitazi et al., 2014	Dietary advice uncontrolled trial	8 children (f = 4, m = 4)	Paediatric Rome III; all IBS subtypes included	Low FODMAP	-	Responders: Greater richness and diversity compared at baseline compared with non-responders. Greater abundance of *Bacteroides*, unclassified Ruminococcaceae and *Faecalibacterium prausnitzii* at baseline compared with non-responders.	[68]
Halmos et al., 2015	Crossover feeding RCT	27 (f = 21, m = 6)	Rome III, all IBS subtypes included	Low FODMAP	Typical Australian diet	No baseline microbiota differences identified between responders and non-responders.	[70]
Chumpitazi et al., 2015	Crossover feeding RCT	33 children (f = 22, m = 11)	Paediatric Rome III; all IBS subtypes included	Low FODMAP	American childhood diet	Responders: Greater abundances for a range of saccharolytic taxa including within the family Bacteroidoidaceae and order Clostridiales (e.g., *F. Prausnitzii*) compared with non-responders.	[79]
Bennet et al., 2018	Dietary advice RCT	61 (f = 51, m = 10), 33 low FODMAP	Rome III; all IBS subtypes included	Low FODMAP	Traditional IBS diet	Responders: Increased abundance of *Streptococcus*, *Dorea* and *Ruminococcus gnavus* at baseline compared with non-responders. Lower dysbiosis index at baseline compared with non-responders.	[5]
Valeur et al., 2018	Dietary advice uncontrolled trial	61 (f = 54, m = 7)	Rome III, all IBS subtypes included	Low FODMAP	-	Responders: Greater abundance of *Bacteroides fragilis*, *Acinetobacter*, *Ruminiclostridium*, *Streptococcus* and *Eubacterium* at baseline compared with non-responders. Lower abundance of *Clostridia*, *Actinomycetales*, *Anaerotruncus and Escherichia* at baseline compared with non-responders.	[77]

IBS, irritable bowel syndrome; RCT, randomised clinical trial.

## Abbreviations

ALT	Alanine aminotransferase
FMT	Faecal microbiota transplant
FODMAP	Fermentable oligo-, di-, monosaccharides and polyols
IBS	Irritable bowel syndrome
PPGR	Postprandial glycaemic response
RCT	Randomised clinical trial
RNA	Ribonucleic acid
SCFA	Short-chain fatty acid
